# Optimal layout of tourist toilets using resilience theory: An empirical study on Dunhua City in ethnic region of China

**DOI:** 10.1371/journal.pone.0251696

**Published:** 2021-05-20

**Authors:** Ling Han, Yeqing Cheng, Zhehao Cui, Guangliang Xi

**Affiliations:** 1 College of Geography and Ocean Sciences, Yanbian University, Yanji, China; 2 College of Geography and Environmental Sciences, Hainan Normal University, Haikou, China; 3 College of Integration Science, Yanbian University, Yanji, China; 4 College of Architecture and Urban Planning, Nanjing University, Nanjing, China; Northeastern University (Shenyang China), CHINA

## Abstract

The provision of adequate and equitable sanitation services is one of the world’s urgent challenges. Optimizing the layout of tourist toilets is key to both meeting the sanitation demand of the visiting public and building an inclusive and civilised society. Nevertheless, the need for a consistent optimization of tourist toilets is overlooked in developing countries, especially in ethnic regions that are highly dependent on tourism. Taking Dunhua, a city in an ethnic region renowned for tourism on China’s border with North Korea as an example, this study enables an optimization framework of a comprehensive tourist toilet layout based on Holling’s concept of resilience by constructing an AHP index, obtaining Point of Interest (POI) data through Python, and aided by GIS visual analysis and Location-allocation (LA) modelling, aiming to support scientific planning and decision making of public facilities in tourist destinations like Dunhua. It also serves as a reference for places of tourism in other countries dedicated to promoting ecotourism and public health.

## 1. Introduction

Adopted by the UN General Assembly in 2015, the United Nations’ 2030 Agenda on Sustainable Development entwined economic, social and environmental targets in 17 Sustainable Development Goals (SDGs) as an ‘indivisible whole’, calling for a global shift to sustainable development [1]. Of these, "providing adequate and equitable sanitation for all" is one of the main goals [2], suggesting it is also one of the world’s urgent challenges. The global outbreak of the COVID-19 highlighted the importance of improving the provision of sanitation services.

**Fig 1:**
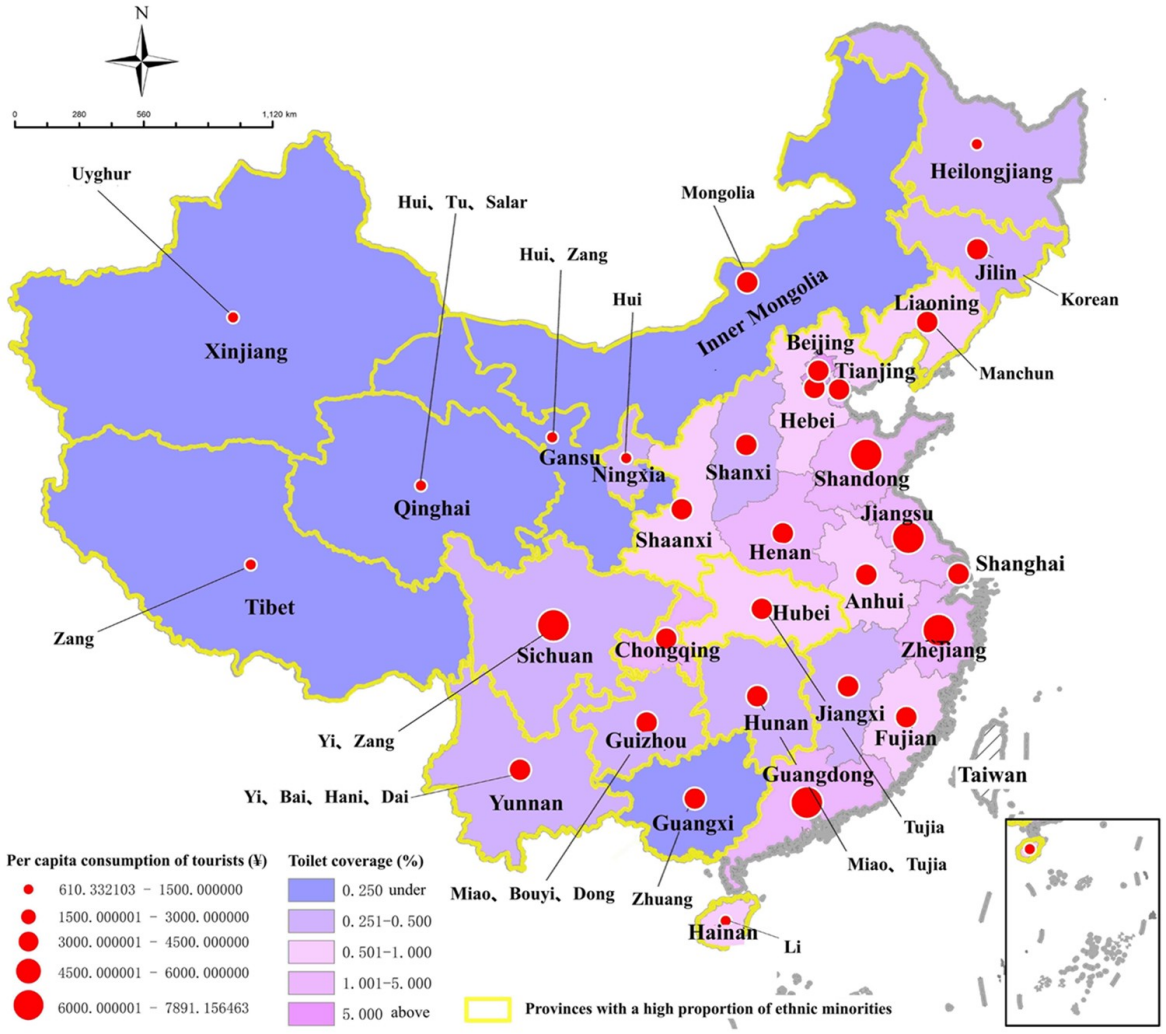
The relationship between the toilet coverage, tourist consumption, and the proportion of ethnic minorities in 2017 (copyright hold by the authors).

Tourist toilet is an indispensable piece of sanitation infrastructures in tourism activities. As "the face of tourist places" and "the measure of human civilization", the value it embodies not only lies in the inherent quality of tourist places and their sustainable and healthy development, but also in the reflection of national civilization image and the comprehensive strength of the country. Nowadays, tourist toilets are no longer limited to public toilets serving tourists in tourist attractions [3], but a comprehensive system including public toilets in various tourism-related places, such as tourism. cities, scenic areas (scenic spots), tourist restaurants and tourist blocks, etc. [4]. However, for a long time, the planning and construction of tourist toilets has been the most prominent weaknesses in sanitation service system and tourism development in many developing countries, especially for ethnic regions (Here, the “ethnics” refers to the non-majority ethnic group(s), they can be indigenous peoples in protected areas of developed countries, but more commonly it refers to ethnic minorities in underdeveloped countries and regions). For instance, in China, since ethnic regions are often highly coupled with underdeveloped regions along the border and regions with rich tourism resources, tourism is often a key way they seek to alleviate poverty and generate income. Its contribution to regional cultural communication, ecological restoration, social stability and national unity is also increasingly being recognized [5, 6]. Nevertheless, the serious backward toilet construction is becoming an important factor restricting the development of tourism in ethnic regions, as well as a shortcoming of China’s social civilization and public health service system. The "Tourist Toilet Revolution" is imperative [7]. Specifically, the tourism industry in China’s ethnic region plays an important role in promoting local economic development, but the toilet coverage (Toilet coverage (rate): each toilet location point is taken as the center, and the surrounding circle with certain radius around that location point becomes the coverage area of that toilet. Divide the accumulated coverage area of all toilets in the area by the total area to get the toilet coverage of a given area) is generally low, and the imbalanced spatial layout of toilets is prominent throughout the country (Fig 1). Tourist toilets should become the focus and breakthrough of tourism infrastructure construction in developing countries, and it should also be an important issue to solve the serious imbalance of social public health services. Therefore, it is of great practical significance to strengthen the research on reasonable layout and optimal configuration of tourist toilets in ethnic regions.

Optimal layout of public infrastructure is one of the core issues of public service supply. The earliest study can be traced back to Weber’s in 1909 on determining the location of a single warehouse to minimize the total distance from each customer [8], while its rapid development began with Hakimi’s research in 1964 [9]. Previously, the objects of the study were concentrated on parks [10], schools [11], industrial zones [12] and places with clean energy charging facilities [13]. Since the beginning of this century, the acceleration of urbanization has exposed the urban problem of the shortage of urban public service supply, making the optimal layout of urban public toilets become an important issue. Sampling surveys, data statistics, and travel population density methods are the most commonly used in quantitative analysis [14–16]. With the development of operations research and computing science in recent decades, facility optimal layout models are also applied in this field [17]. Recent studies have tried and explored GIS technology to optimize the location of public toilets. Quaye, for instance, used GIS and statistical methods to establish a map and mathematical model of the impact of population growth on health infrastructures in densely populated areas, and analyzed, predicted and visualized the number of public toilets [18]. Edilberto created a time-series map through GIS to visualize the changes of health services over time in Parana, Brazil [19]. Feng and Long combined quantitative analysis methods with GIS technology to analyze the rationality of existing layout of urban public toilets and predict its demand in the future [20, 21].

As the penetration of public toilets research in the tourism field, prior studies on tourist toilets mainly focuses on three types: problem-solving, optimal layout, and tourists’ perception and evaluation. Gong and Huang analyzed the existing problems and their causes in the overall construction of tourist toilets, and put forward corresponding solutions [22, 23]. Optimal layout studies are mainly carried out from two aspects of macro planning and micro design. Zhang et al. discussed the general principles of location selection of tourist toilets in tourist attractions and the processing techniques of their interior space layout from the perspective of planning and design [24–27]. In tourist perception and evaluation studies, Sun et al. evaluated the quality of tourist toilet construction in urban areas such as Suzhou and Changsha as well as representative scenic spots in Chongqing and Shandong Province by adopting structural equation modeling method and AHP and IPA quantitative analysis methods [28–31], which strengthened the reliability of the conclusions and found that reasonable layout and site location were crucial for improving tourists’ satisfaction with tourist toilets.

Moreover, in the “SDGs”, ‘build resilient infrastructure’ is also one of its goals. “Resilience” was originally introduced by Holling in 1973 as a concept to help understand the sustainability of the ecosystem in its original state [32]. Since 2000, the concept of resilience began to replace the concept of "sustainable development" and became a new theoretical focus. It can be deconstructed into ecological resilience, engineering resilience and social resilience [33]. Sociologists, especially human geographers, widely adopted the resilience theory to discuss how to foster resilience of cities in face of the acceleration of global climate change and urbanization, and how to cope with socioeconomic and political uncertainty risk through the layout of green infrastructure, the construction of models, and rational land use planning, etc. [34, 35]. Nowadays, the concept of resilience becomes a new paradigm in tourism research. Chen et al. proposed the concept and research framework of tourism socio-ecosystem, believing that resilience planning, resilience identification and resilience promotion should be the focus of tourism system research [36, 37]. Tourism activities are a continuous dynamic process with diversity and complexity, changes in the number of tourists will cause different demand for tourist toilets, which objectively requires the layout of tourist toilets should dynamically adapt to the changing needs of tourist destinations, rather than be a product of a one-time rigid planning [38]. The embedding of "resilience" is able to meet the demand, so as to promote the effective adaptation and transformation of tourist toilets under dynamic external influences, and avoid extreme phenomenon such as waste of resources or excessive pressure on resources.

Several key concerns can be raised about this research. Although the meaning of tourist toilets has been extended, the scope of the research is still confined to toilets in scenic spots, and lacks comprehensive attention to tourist toilet system, and even less concern shown to those in ethnic regions. Among the facility layout models, LA model is the most widely used one, but this kind of analysis and other traditional quantitative methods as well as GIS technology are rarely used in the study of tourist toilets. As a result, these studies are still dominated by qualitative and descriptive methods, and the adjustment of the layout of tourist toilets is mostly a top-down supply-driven planning, so the fairness and operability of the conclusions are insufficient. Furthermore, to our knowledge, so far, resilience theory has not been employed to the study on optimal layout of tourist toilets. Static study focusing on a single time section reduces the overall social benefits of the layout.

Through combining AHP method and LA model, this study made a deep use of GIS’s comprehensive abilities of geostatistics, spatial analysis and model solving to improve the research method and conduct an empirical study on Dunhua, a tourism city within the border ethnic region of Jilin Province, China. A spatial database of tourist toilets was established and visualized, as well as a systematic optimal layout scheme of different types, specific locations and grade classification aspects of tourist toilets was put forward from the perspective of both urban area and scenic area. Resilience theory was introduced into the research, offering a creative insight for promoting the balance, convenience and flexibility of the tourist toilet supply.

## 2. Materials and methods

### 2.1 Study area

**Fig 2:**
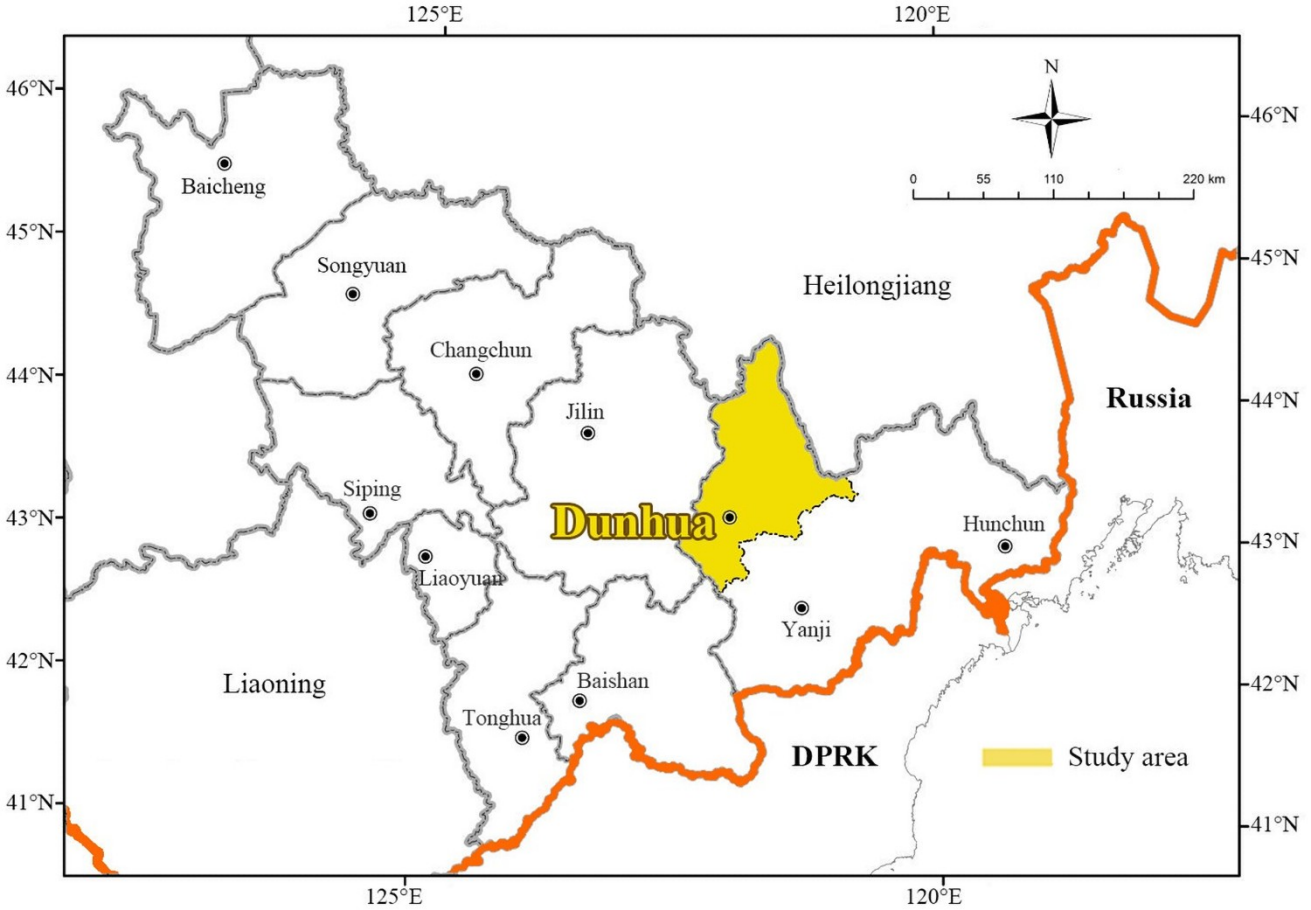
Study area (copyright hold by the authors).

Dunhua City is located in Yanbian Korean Autonomous Prefecture, an ethnic region in Northeast China’s Jilin Province that borders North Korea. It is adjacent to China’s Heilongjiang Province to the north, the coastal border area of Russia to the East, and DPRK’s Hamgyong and China’s Liaoning Province to the south. Located in the hinterland of Changbai Mountain, the city is rich in natural resources cultural heritage and national scenic areas. It is a key area for tourism development in Jilin Province and a famous garden city in China (Fig 2).

**Fig 3:**
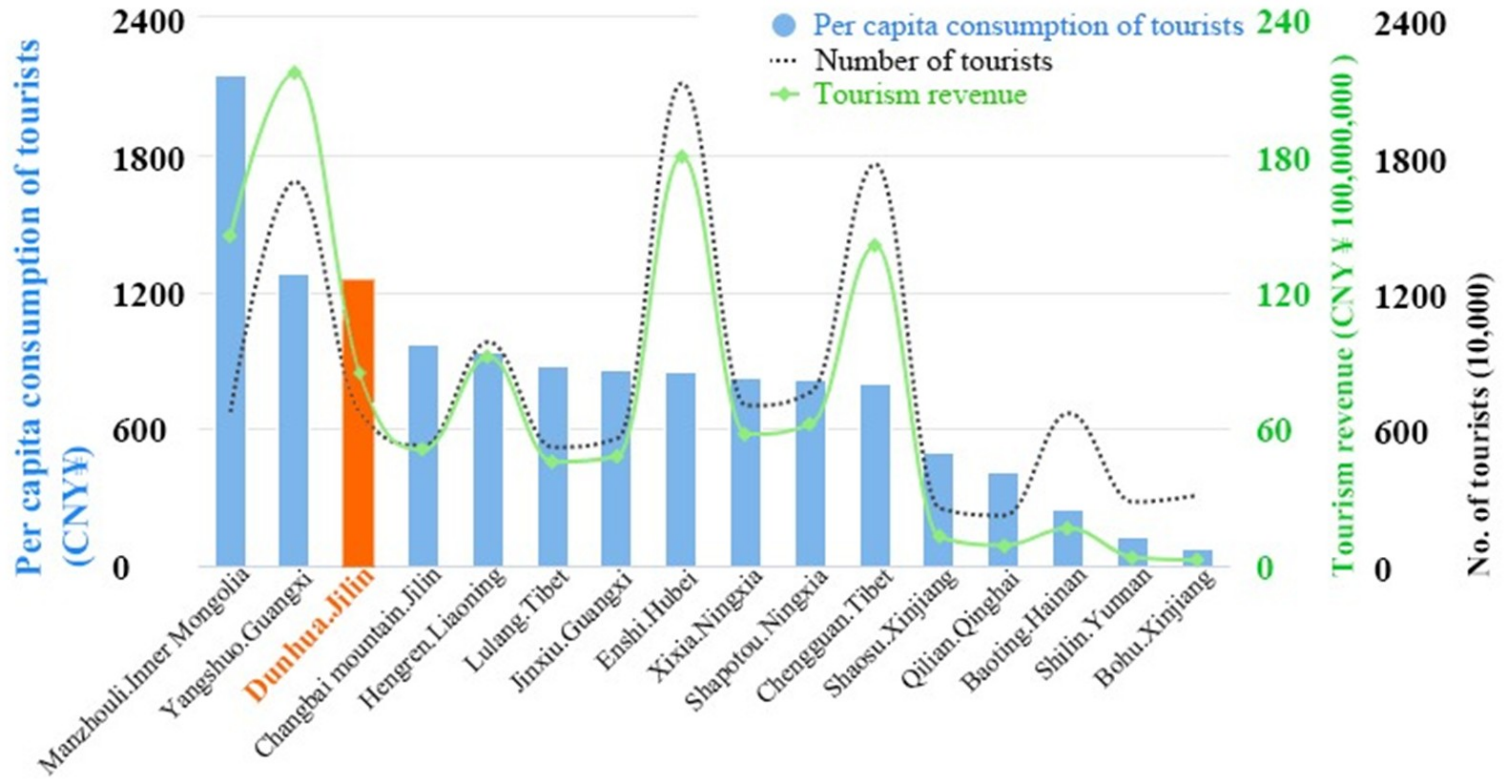
Tourism data of the 16 national ethnic regions in 2019 (copyright hold by the authors).

Based on the above situation, Dunhua has a wide tourism market at home and abroad. In the past five years, the average annual growth rate of tourism person-times has reached 37%. In 2018, 6.73 million domestic and foreign tourists were received, and tourism revenue came to $1.28 billion, accounting for 24.5% and 16.3% of the total tourism person-times and tourism revenue across the ethnic region. At present, Dunhua City has formed an overall tourism development pattern based on the urban area, linking with four key scenic areas, namely the Liuding Mountain Tourist Area (LDM), Laobai Mountain Ecology Tourist Area (LBM), Yanming Lake Hot Spring Resort (YML) and Hancong Ridge National Forest Park (HCR). The latest statistics show that tourists to these four key scenic areas accounted for 88.2% of the total tourists in the city’s A-level scenic areas of 3.011 million, among which LDM was 1.857 million, which was the main tourist destination for tourists. In addition, among the first 71 National Tourism Demonstration Zones publicized by the Ministry of Culture and Tourism in 2019, 16 ethnic regions were on the list, while Dunhua City is the only one selected among the ethnic regions in Jilin Province, and the level of per capita consumption of tourists that contributes significantly to the local economy ranks third among those regions (Fig 3).

### 2.2 Data sources

#### 2.2.1 Population data

The urban resident population and area data were obatained from National Bureau of Statistics of China (http://www.stats.gov.cn/tjsj/ndsj/) and the <Dunhua Statistical Yearbook (2015–2019)> on the official website of Dunhua Municipal People’s Government (http://www.dunhua.gov.cn/sj_2154/).

#### 2.2.2 Tourism data

Tourism data such as the number of tourists, tourism revenue and tourist toilets came from <The Yearbook of China Tourism Statistics (2017)> [39] and the official website of Bureau of Tourism of Dunhua (http://www.dunhua.gov.cn/sq_2085/dhly/). Other data such as the toilet coverage rate were calculated based on statistical yearbook data or GIS technology.

#### 2.2.3 Geomatic data

The maps of administrative division, land use, transportation and scenic area planning were powered by the People’s Government of Dunhua, China, while the remote sensing images were obtained through Geospatial Data Cloud Platform of the Computer Network Information Center of Chinese Academy of Sciences (http://www.gscloud.cn).

#### 2.2.4 POI data

Amap (https://www.amap.com/) is one of the leading Internet map and navigation service providers in China, where POI data in the urban area of Dunhua City were crawled from. In addition, field surveys were conducted on the tourist toilets in the streets, communities, residential areas and scenic areas et al. to ensure the accuracy of the data and results.

### 2.3 Research methods

#### 2.3.1 AHP index

**Fig 4:**
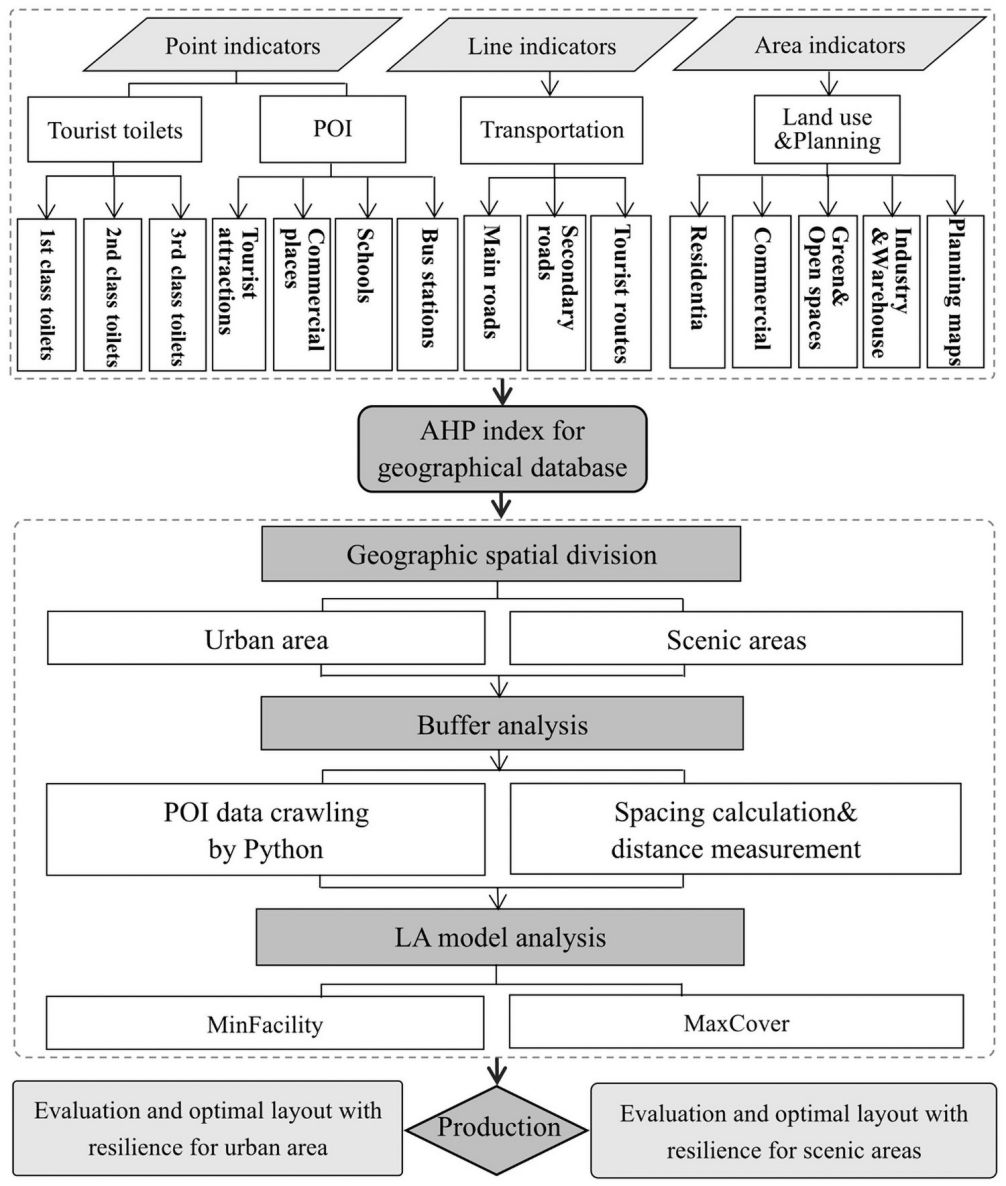
Flowchart (copyright hold by the authors).

The analysis framework (Fig 4) was first based on the Analytic Hierarchy Process (AHP). A set of the layers including target, criterion and indicator was defined to meet a siting objective. The indicator layer is a composite of point, line and surface elements. Considering the layout of tourist toilets in urban area should not only consider the needs of tourists, but also serve local residents, the influencing factors are more diverse and complex. Therefore, in addition to collecting tourist toilet points according to different classes, the selection of "point" indicators was based on the principle of large population flow. This study also applied Python technology to crawl, process and classify four types of POI data of bus stops, consumption places, schools and tourist attractions, representing the request locations of tourist toilets. Finally, we collected a total of 3726 pieces of information points, including 82 bus stops, 3555 consumption places, 82 schools and 7 tourist attractions, which effectively reflect the needs of residents and tourists. Combined with the "line" and “area” indicators, the AHP index system of urban tourist toilets was completed. Tourist toilets in scenic areas are planned for serving tourists, so the system was constructed based on scenic spots, tourist routes, functional areas, and tourist capacity, etc.

#### 2.3.2 Buffer analysis

To evaluate the coverage gap of current tourist toilets in terms of quantity, class and spatial distribution, the study employed the spatial analysis function of GIS to conduct buffer analysis on current tourist toilets, calculate the coverage level of tourist toilets and visualize it. Then, based on remote sensing images and transportation maps, a transportation database was established, which laid an important foundation for model analysis and site selection optimization.

Notably, this effectively improved the efficiency of tourist infrastructure and facilitated a more “resilient” structural system. That was, increased the resilience by determining the number and location of resilient tourist toilets. In this paper, mobile toilets served as resilient tourist toilets since they have the characteristics of movable, composable, designable, convenient in transportation and management, and also have the advantages of flexible, eco-friendly, adaptable to the dynamic nature of tourism activities. Although there was no construction standard for mobile toilets, they were considered as class II toilets due to their higher internal configuration than ordinary fixed toilets. Resilient toilets were mainly located in places with large tourist flow during peak season to alleviate the external impact of tourists. The reasonable number and specific location were determined through the simulation of LA model by calculating the elastic range of the number of tourists and the elastic range of the number of tourist toilets in peak-off season.

#### 2.3.3 LA model

Adheres to mathematical programming, the location-allocation (LA) is to “locate a multiple number of facilities and allocate the demand served by these facilities so that the system service is as efficient as possible” [40]. The analysis principle is to treat the facilities that need to be arranged as service providers, which are represented by points, i.e. "facility points", and treat the users of services as demanders, which are represented by points, i.e. "demand points" [41]. In the process of LA analysis, different models will be formed through the combination of different facility types and different criteria. We tried to simulate a better solution layout of tourist toilet in the study area using the Minimum facility point model (MinFacility) and the Maximum Coverage (MaxCover) model, aiming to minimize the distance to tourist toilets for citizens and tourists to provide more convenient health service facilities, and enable tourist toilets facilities to cover as many demand points as possible within a certain service radius. The purpose is to minimize the distance between citizens and tourists to reach the facilities while also saving money, that is, to allow as few facilities as possible to cover as many demand points as possible within a certain service radius.

The first location analysis is based on the MinFacility model. This step aimed to select as few “facility points” as possible from all the candidate facility locations, so that the maximal facility “demand points” are within the maximum service radius of these facilities [42]. The model can automatically calculate the minimum number of tourist toilets that can basically meet the demand. In this step, the minimum impedance matrix can be constructed first to save time cost, and then it can be formulated as:

$$Lab=Dab+\sum_{n-1}^{n}Xn$$ (1)

where Lab represents the total resistance of the node pair (a, b) on the path, Dab represents the impedance of all sections of the node pair (a, b) on the path, and Xn refers to the impedance spent on the section to the nth node (it can be expressed in terms of time or in terms of length, which is the delay time multiplied by the velocity).

The second location analysis is based on the service scope of current facilities. By removing the first location sites that overlap with the current toilets and those that have been covered by the current tourist toilet service range, the second location sites were obtained.

The final location of tourist toilets is determined based on the MaxCover model. The purpose of the MaxCover Model is to select a given number of spatial locations of facilities from all candidate “facility points” so that the maximal “demand points” are within the maximum service radius of the facilities [43]. This step is mainly used to compare and optimize the effect. On the premise of referring to the number of tourist toilets predicted by the population distribution analysis method, we finally determined the number of toilets in the way of artificial correction to avoid resource waste that may be caused by the system’s automatic selection. The calculating process can be formulated as:

$$\text{max}=\sum_{a=1}^{n}QaYb$$ (2)

$$Q.T.\left\{ \begin{array}{l} \sum_{a=1}^{m}CabXa-Yb\geq 0\\ \sum_{a=1}^{m}Xa=D\\ XaYb=0,or1(a=1,2\cdots n,b=1,2\cdots m) \end{array}\right.$$ (3)

where, Xa, Xb is the decision variable. If Xa = 1, the facility is configured at the demand point a, otherwise Xa = 0; if Yb = 1, the demand point b is covered within the effective service radius of the facility, otherwise Yb = 0; Qa is the comprehensive layout index of the demand point to the facility point; Cab is the binary value coefficient, when the distance Lab from the demand point a to the facility point b is within the effective service radius R, Cab = 1, otherwise Cab = 0; D is the specified number of facility layouts.

## 3. Results

### 3.1 Current layout of tourist toilets

#### 3.1.1 Urban area

**Fig 5:**
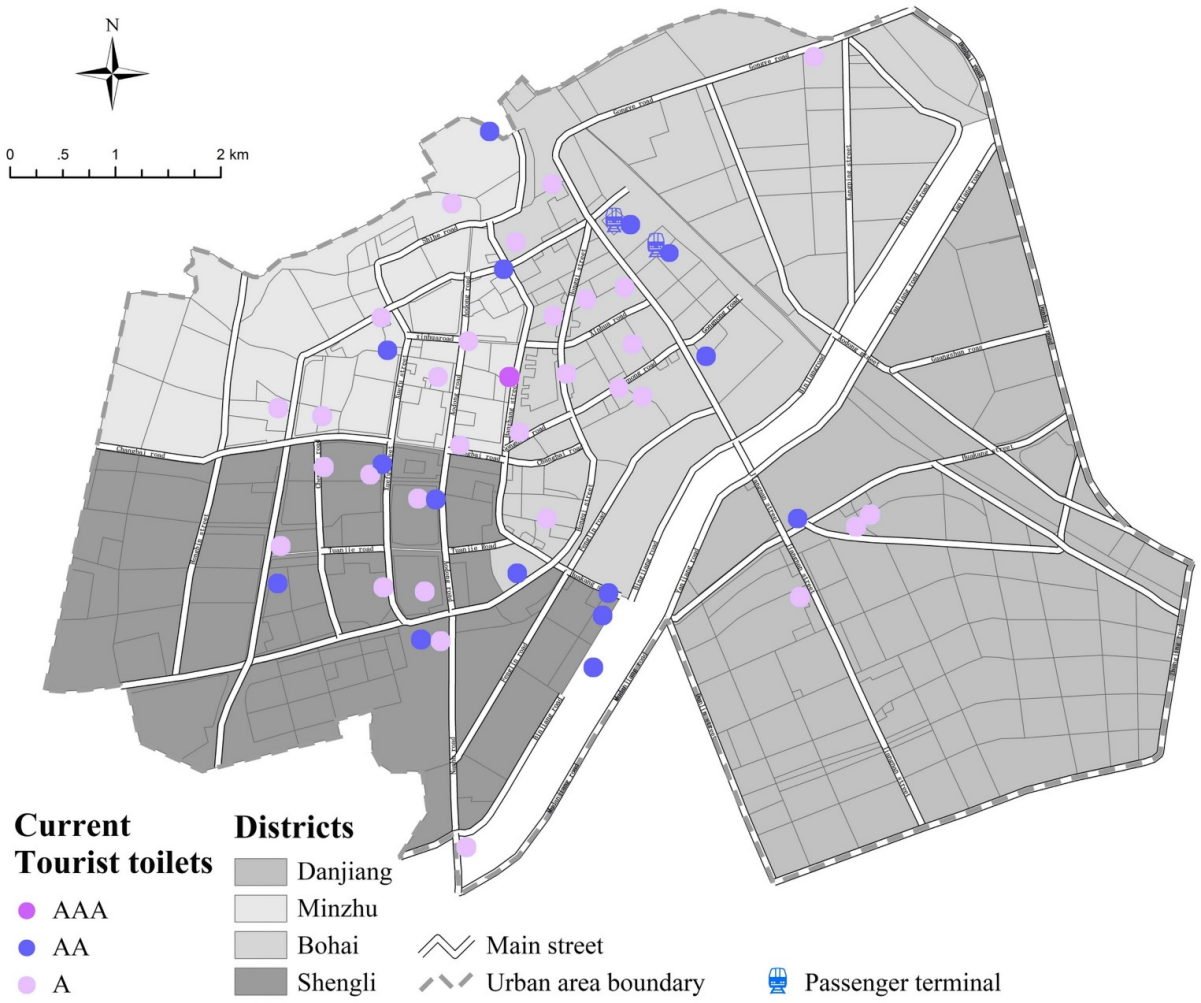
Layout of current toilets in each districts of urban area (copyright hold by the authors).

The urban area of Dunhua City is 47.17 km^2^, with a total of 189,806 people and 46 tourist toilets. <Standard of Setting of Environmental Sanitation Facilities (CJJ-27-2012)> regulates that there should generally be set one public toilet in urban areas for every 2500–3000 people [43]. Therefore, 65–76 fully open tourist toilets should be set in the urban area of Dunhua City, and 19–30 public toilets should be added. To be specific, the prescribed number of toilets in Minzhu district should be 11–13, but the actual number is 11. There should be 18 to 22 toilets in Shengli district, but actually there are 16. Bohai district should have 20–23 toilets, actually 15, and Danjiang district should have 16–20 toilets, but actually 4. The results showed that the number of tourist toilets in urban area do not reach the standard except for Minzhu district. Especially in Danjiang district, a new urban area to the south of Mudanjiang, where the number is seriously insufficient (Fig 5).

In terms of the class of tourist toilets, <Standards for Planning of Urban Environmental Sanitation Facilities Planning (GBT 50337–2018)> [44] regulates that: (1) In urban built-up area, 65% of toilets should be Class II (AA) or above. (2) In CBDs, parks, squares and other landmark areas, class I (AAA) toilet should be installed. (3) Important roads, large-scale venues, municipal offices and other central city public activity areas should be equipped with class I or class II (AA) toilets. (4) Toilets of class II should mainly be arranged in general roads and residential areas, while class III (A) toilets could be installed appropriately. (5) Secondary and branch roads and sparsely populated areas are mainly set up with class III toilets. However, the total number of toilets of class I, class II, and class III in the urban area are 1, 15 and 30 respectively, and the proportion of class II or above is only 35%, which is far lower than the national standard. Particularly, the number of toilets of class II or above in central city public activity areas such as the Hanzhang Street, Aodong Street and Xuefu Street are seriously insufficient, while toilets of class III exceed the number of standard.

According to the service radius standards in GBT 50337–2018 [44], in accordance with the general principles of ’taking big instead of small’, ’integering to decimal point’ and ’taking upper limit’ in tourism cities, combined with the regional characters, the administrative level and other situations of the city, this paper defined the local standard for the service radius of the tourist toilets in the urban area That is, 200m in land of Public Service Facility (A) and Green and Open Space (G),150m in land of Commercial (B) and Transportation (S),350m in land of Residential (R),500m in land of Industrial (M), Warehouse (W) and Utilities (U).

**Fig 6:**
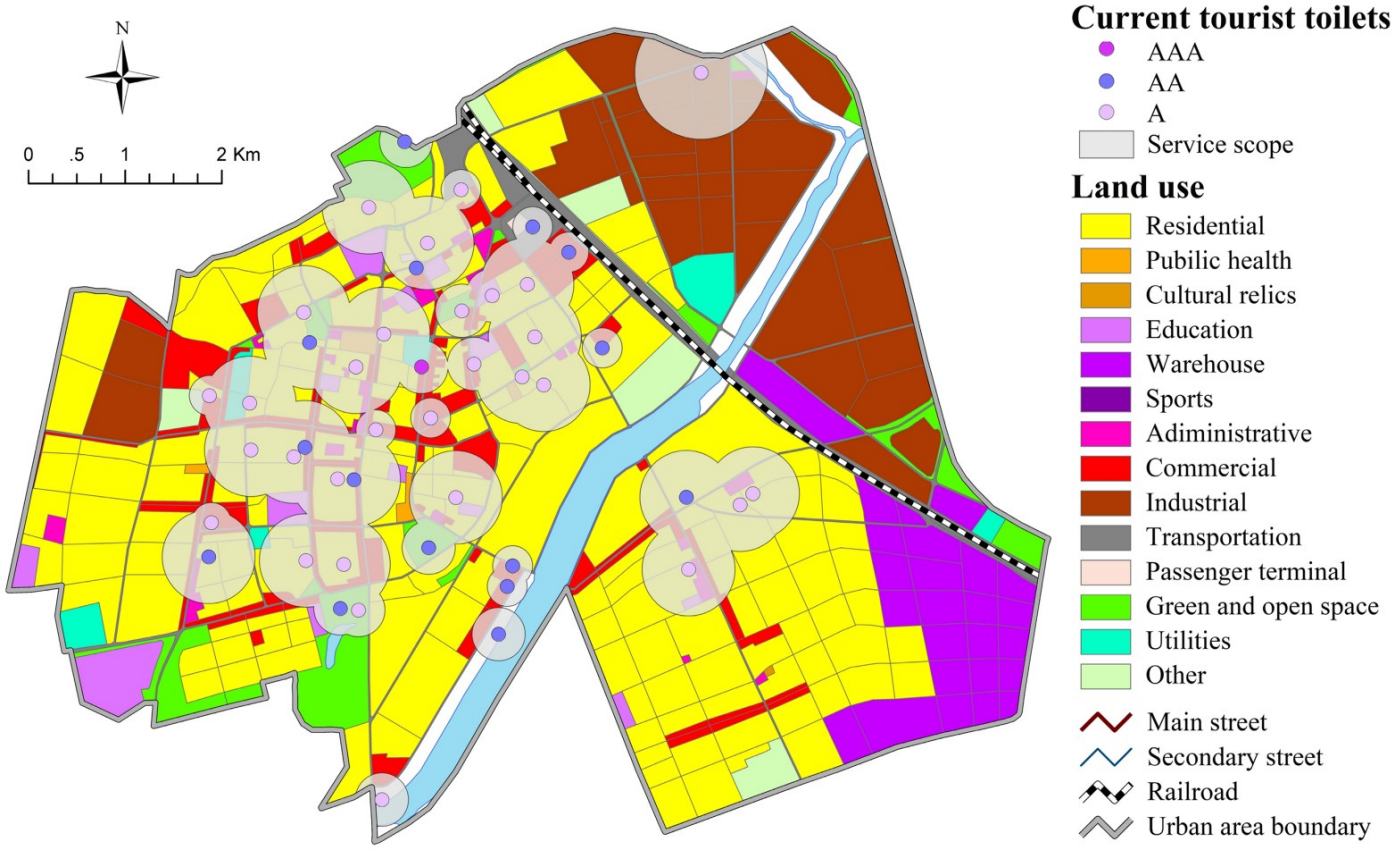
Service scope of current toilets in context of land use (copyright hold by the authors).

Fig 6 shows that through buffer analysis, the service scope of existing toilets in the urban area is 15.42 km^2^, with a coverage rate of 33%. In the transport hub station with the highest population density, the toilet coverage can reach 87%, which is relatively convenient for citizens and tourists. However, it was suggested that the resilience of this area should be increased during the tourism season. In the high-density concentration areas represented by Changbai Road, Tuanjie Road, Aodong street and Hanzhang street, although the number of toilets is large and dense, it was found that some important areas are not covered by their service scope. Green spaces and squares are one of the main venues for citizens and tourists. The requirement in GB 50337–2018 that "CBD, parks, squares and other landmark areas should be equipped with Class I toilets" suggests that such areas are also key areas for toilet layout, but their toilet coverage is low, standing at 32%. Additionally, the toilet coverage rate in industrial and warehouse land is obviously low.

#### 3.1.2. Scenic areas

**Fig 7:**
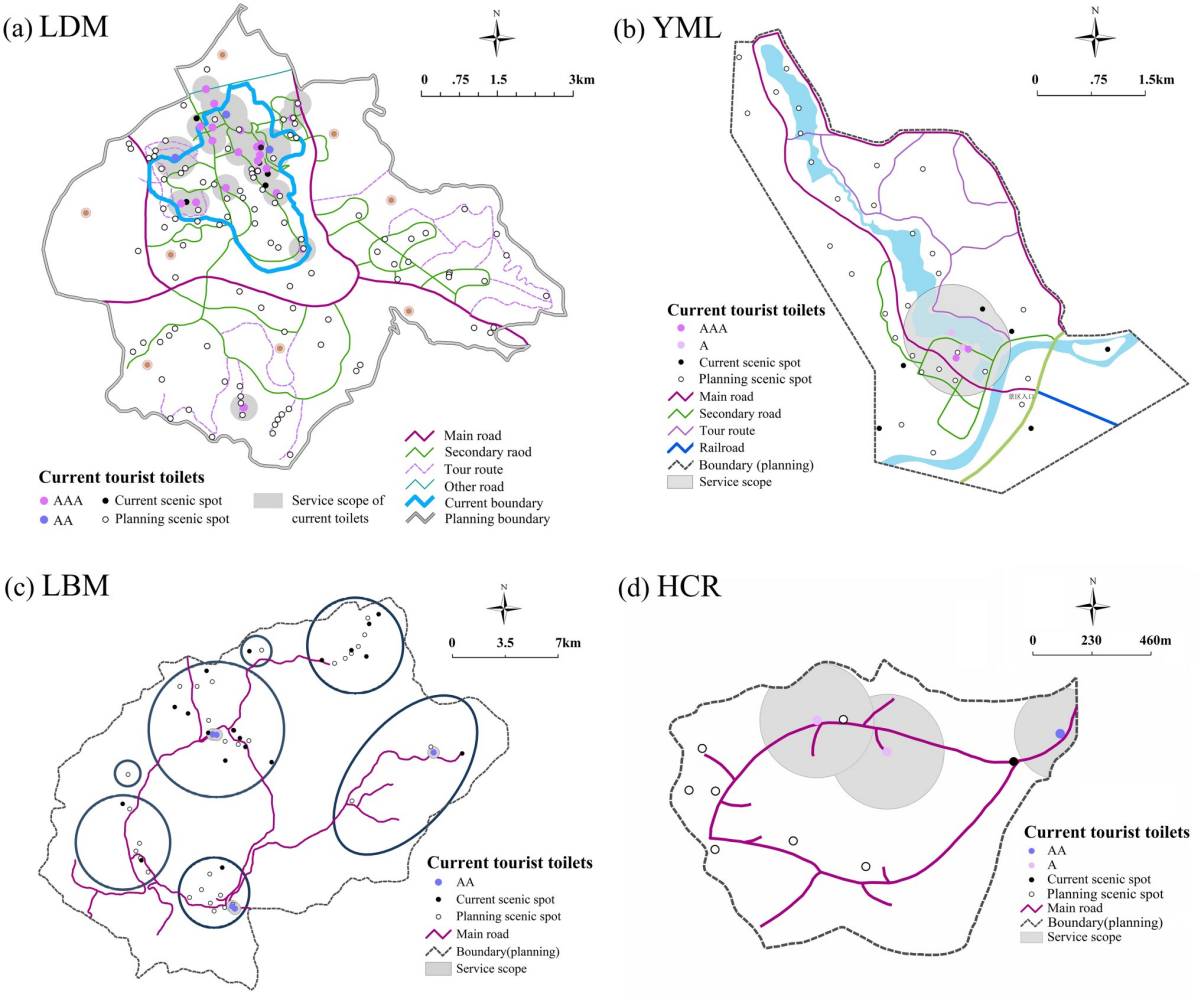
Current layout of tourist toilets in key scenic areas (copyright hold by the authors).

<Classification and Evaluation of Tourist Toilets (GBT 18973–2016)> stipulates that the maximum distance of the tourist toilets of class III, class II and class I should not exceed 1000m, 800m and 500m respectively [45]. According to this standard, the buffer zone analysis was carried out on tourist toilets in the four main scenic areas of LDM, YML, LBM and HCR, as shown in Fig 7. Fig 7(a) shows that LDM has a total area of 45.3km^2^, 5.9km^2^ of which is current construction area. It has 19 tourist toilets, of which 16 are class I and 3 are class II. The service scope of these toilets is 2.7km^2^, which covers 46% of the scenic area and 100% of all the scenic spots, indicating that it has a better condition in the number and quality of tourist toilets However, there are a total of 102 scenic spots in the planning area of the LDM, only 19 of these are served by the current tourist toilets, with a coverage rate of 9%, which is far from meeting the needs for future tourism development. At present, there are only 4, 5 and 3 tourist toilets in YML, LBM and HCR scenic areas. Compared with LDM, the tourist toilets are in poor condition (Table 1).

**Table 1:** Service data regarding tourist toilets in key scenic areas.

Name	Level	Size (km^2^)	Quantity	Coverage of Tourist Toilets		
				Scenic Area Coverage Rate (planning area)	Scenic Spot Coverage Rate* (planning area)	Scenic Spot Coverage Rate (current area)
LDM	AAAAA	45.3	24	9%	19%	100%
YML	AAAA	10.9	2	8%	22%	17%
LBM	AAA	47.9	5	5%	7%	5%
HCR	AAA	5.4	4	30%	12%	0

*Scenic spot coverage rate of toilets refers to the ratio of the number of scenic spots covered by the toilet buffer service area divided by the total number of scenic spots in the study area, it can directly reflect the effectiveness of the service scope of the tourist toilets.

### 3.2 Optimal layout of tourist toilets

#### 3.2.1 Urban area

**Fig 8:**
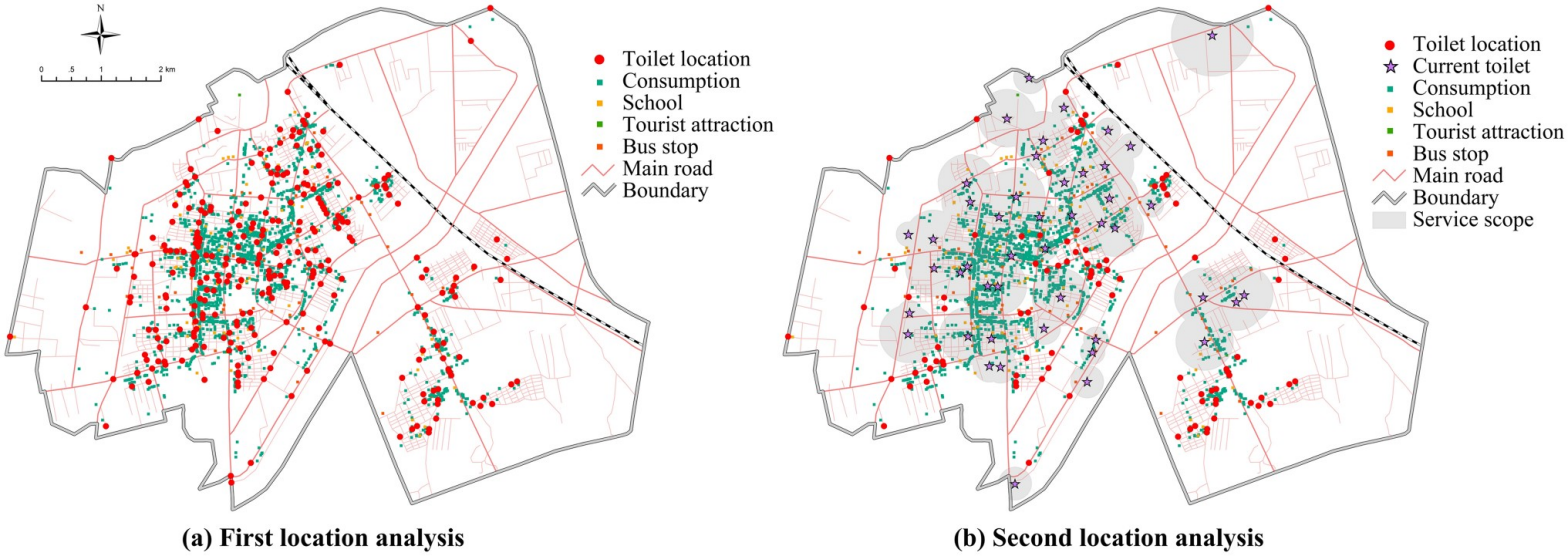
The first and second location analysis process (copyright hold by the authors).

In the first location analysis, considering factors such as traffic data and POI distribution, from the perspective of the physical condition of the tourists (regardless of age), the running speed, the longest supporting time and the impedance interruption was set as 3.4km/h, 15min and 800 respectively, and then we obtained 284 sites by using MinFacility model. In order to reduce the waste of public resources, the points within the service scope of current toilets were eliminated and 95 secondary sites were obtained (Fig 8).

**Fig 9:**
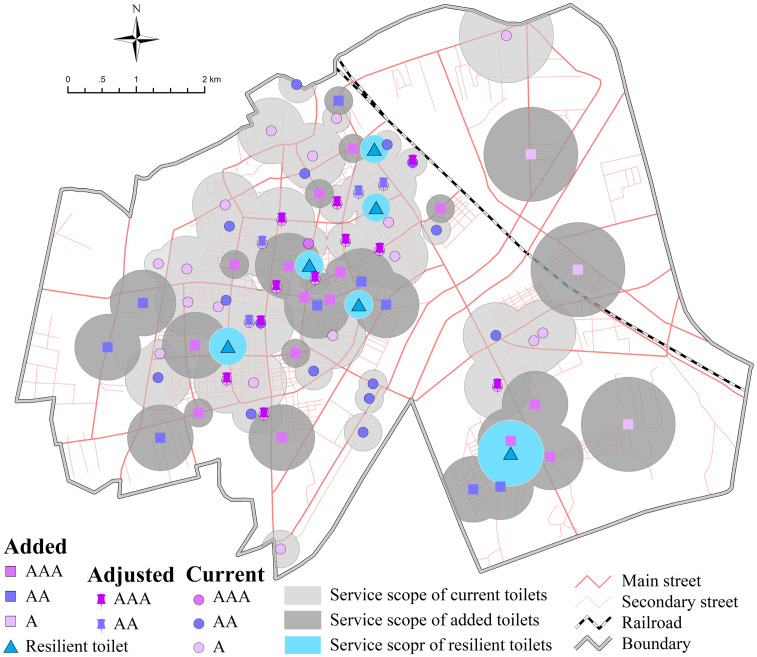
Optimal layout scheme for tourist toilets in urban area (copyright hold by the authors).

Furthermore, we simulated the specific location of tourist toilets with maximum coverage demand using the MaxCover model. Finally, 26 new sites were selected based on the number of new toilets (19–30) calculated by the population distribution analysis, the real situation of the survey and the future development trend of the urban area. In addition to the existing 46, there are a total of 72 fixed tourist toilets (Fig 9).

Statistics showed that the number of tourists in the peak and off-season was 4.26 million and 2.47 million in 2018 respectively, who mainly came from the adjacent areas of Yanbian Prefecture and the Northeast China. Tourists stayed on average for 1 day, so the estimated tourist capacity is 14000 to 24000 people per day. Based on the CJJ-27-2016 standard, it is estimated that 5–10 resilient toilets need to be added. Then, through statistics on the distribution density of POI influence factors, 6 resilient toilets were arranged in the areas with the highest traffic accessibility and population flow density, and they will be opened according to different seasons and time periods. In addition, it is necessary to upgrade or transform 15 toilets in public activity areas of central city, so that the number of tourist toilets of class I, class II and class III reaches 27, 25 and 20 respectively, with the number of class II or above toilets accounting for 72% of the total. Consequently, the service scope of tourist toilets will expand to 30.29 km^2^, with a coverage rate of 64%.

#### 3.2.2 Scenic areas

The reasonable distances between tourist toilets in scenic areas were determined on the basis of the general physiological endurance time, the average walking speed of tourists of different ages, and the standards in relevant documents and literature [24, 45]. The average walking speed were obtained from the average walking speed of 10 randomly selected young, middle-aged and elderly tourists over the same section of a road, while the average distance of tourist toilets required by tourists of different ages relied on the average value calculated from the minimum and the maximum physical endurance time (7–15 minutes) (Table 2).

**Table 2:** Calculation of distance between tourist toilets.

Type of Tourists	Average Walking Speed (v)	Physiological Endurance Time (t)	Calculation Formula	Average Distance
Young (18–40 years old)	3.4km/h	7min	0.95m/s×60×7 = 396m	623 m
		15min	0.95m/s×60×15 = 850m	
Middle aged (40–60 years old)	3.0km/h	7min	0.83m/s×60×7 = 350m	550 m
		15min	0.83m/s×60×15 = 750m	
Elderly (over 60 years old)	2.5km/h	7min	0.7m/s×60×7 = 292m	458 m
		15min	0.7m/s×60×15 = 625m	

**Fig 10:**
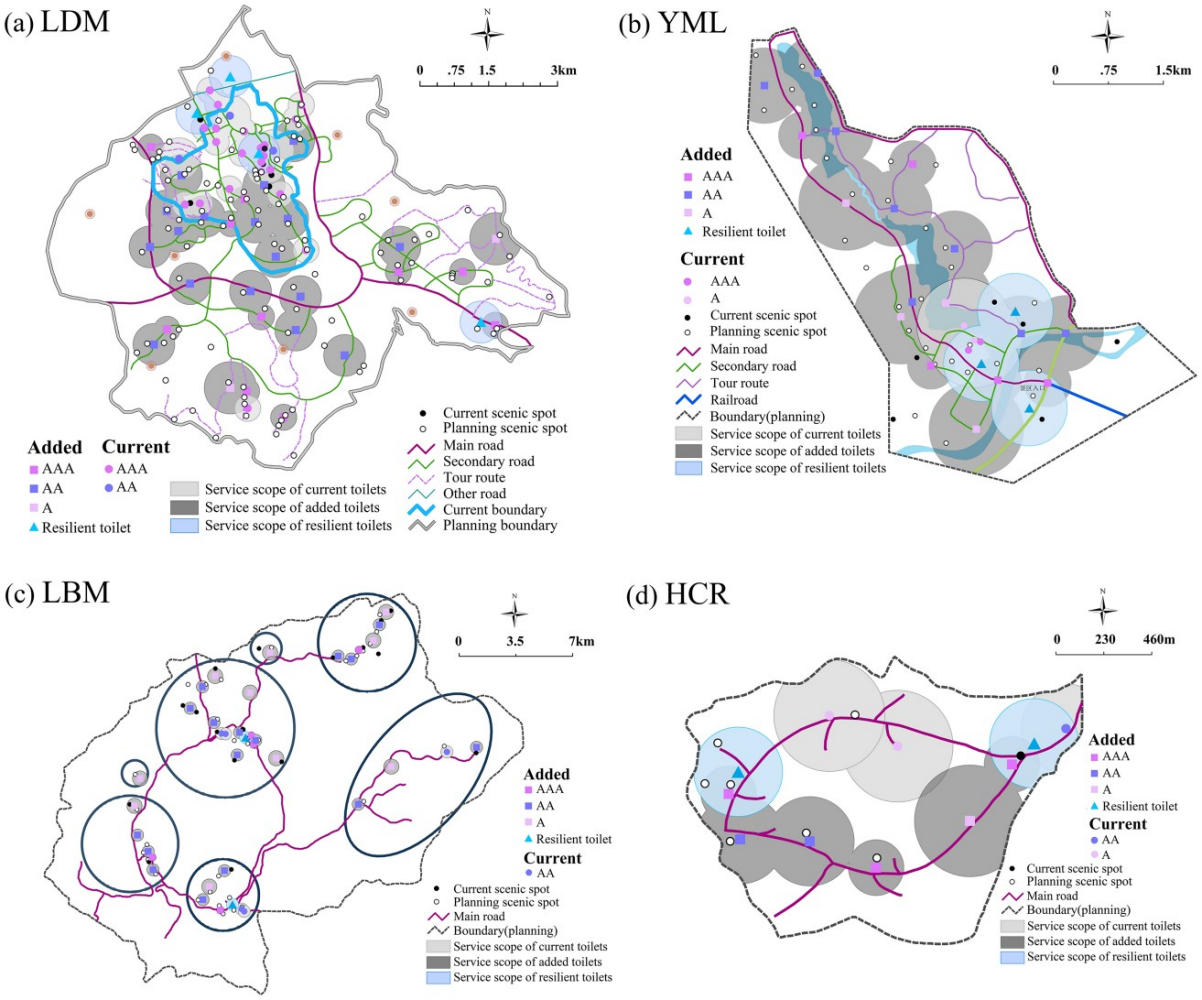
Optimal layout schemes for tourist toilets in scenic areas (copyright hold by the authors).

Considering that China has entered an aging society and the high frequency of urination among the elderly makes them more dependent on toilets than other types of tourists, the main reference standard for determining the distance between tourist toilets should be the walking speed and endurance time of the elderly. Therefore, in principle, the distance between toilets in dense tourist areas is set at 400m, and in general areas at 600m. By integrating the existing data, the optimal layout schemes for the location selection of tourist toilets in the four key scenic areas were proposed (Fig 10).

The specific scheme of each scenic area is as follows: 1) In LDM, 8 new toilets should be added in current scenic area, increasing the coverage rate from 45% to 68%, which will make up for the gap of toilet service scope in the current area, while 29 new toilets should be set in the planning area, including 4 resilient toilets. Thus, the total number of fixed tourist toilets will reach 53, including 31 class I, 20 class II, 2 class III. The service scope of toilets will reach 14.7km^2^, the coverage rate will increase from 9% to 33%, and the scenic spot coverage of toilets will reach 94%. 2) In YML, 17 new toilets should be added, with 3 resilient toilets, and the total number of fixed toilets will reach 21, including 7 class I, 8 class II and 6 class III. The service scope of toilets will reach 5.75km^2^, the coverage rate will increase from 8% to 53%, and the scenic spot coverage of toilets will reach 89%. 3) In LBM, 31 new toilets should be added, with 2 resilient toilets, and the total fixed toilets will reach 36, including 5 class I 5, 21 class II and 10 class III. The service scope of toilets will reach 19.3km^2^, the toilet coverage will increase from 5% to 40%, and scenic spot coverage of toilets will reach 91%. 4) In HCR, 6 new toilets should be added, with 2 resilient toilets, and the total number of fixed toilets will reach 9, including 3 class I, 3 class II and 3 class III. The service scope will reach 4.1km^2^, the toilet coverage will increase from 30% to 77%, and the scenic spot coverage of toilets will reach 100%. The field survey also found that during the peak tourist season, the queuing time of toilets reached at least 3 minutes at the entrance, exit, parking lots and other crowded areas. Given this, this paper took the load caused by sudden flows of large number of people at certain times of year into consideration, and located resilient toilets near the entrances, parking lots, and popular scenic spots, so as to improve the resilience of tourist toilets, especially during holidays and peak seasons.

## 4. Discussion

The results of these analysis identified that the layout of tourist toilets in urban area presents the characters of quantity shortage, lower class, limited service scope, unbalanced overall layout caused by obvious extreme agglomeration of the old and new urban areas. This situation fails to meet ordinary demand from citizens and lacks resilience during the tourism season. Additionally, the gaps in the toilet service in important areas have exposed the unreasonable level setting and location of tourist toilets, which brings negative impact on the city’s tourism image. Since the areas to the west of the Danjiang district are the main direction of urban expansion, and close to the core scenic area of LDM, the demand for tourist toilet service is increasing gradually. Nevertheless, its planning and construction also fail to keep up with the development pace of the city. For scenic areas, due to the fact that LDM is the only national 5A-level scenic spot in the this ethnic region, a serious tilt in resource input inevitably appeared. This also lead to homogenization phenomenon in the layout of its tourist toilets. The over dense arrangement of Class I toilets can easily lead to a waste of resources, and has a potential impact on the surrounding environment. Compared with LDM, the situation of tourist toilets in YML, LBM and HCR is more problematic, including seriously insufficient quantity, low class, small service scope, unreasonable layout, resulting polarization between scenic areas and uneven distribution of resources.

**Fig 11:**
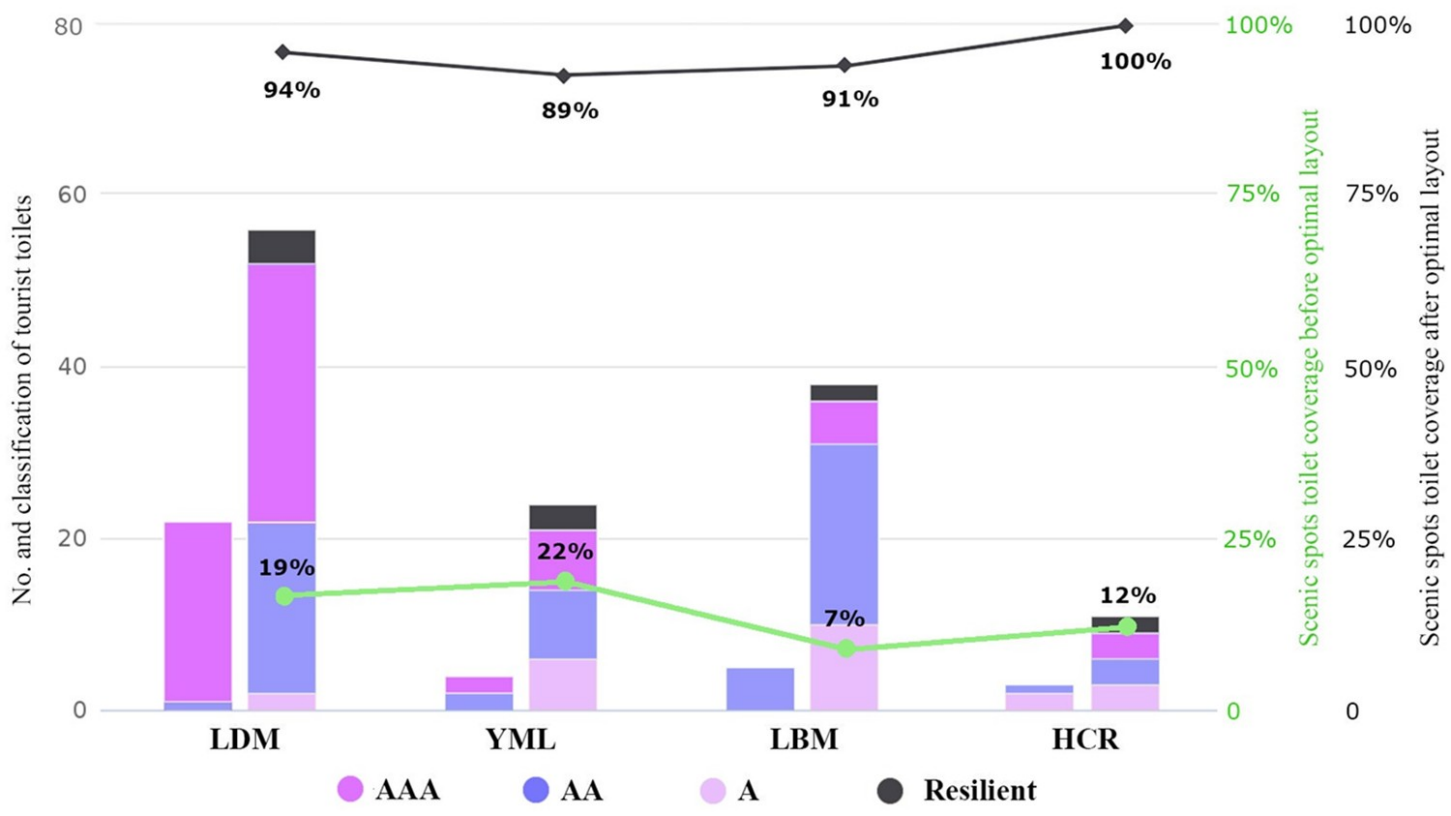
Statistics of tourist toilets in scenic areas before and after optimization (copyright hold by the authors).

Our study extended previous research that LA modeling can effectively avoid wasting resources and provide a scientific basis for the reasonable layout of tourist toilet facilities with different types and different grade classification from the two dimensions of urban and scenic areas. Based on this, it was proposed to renovate and upgrade 15 existing toilets, add 26 new fixed toilets and 6 resilient toilets in the urban area, thereby increasing the toilet coverage from 33% to 64%, which meets the requirements of the national setting standard of urban environment and sanitation facilities. In the scenic areas, the average scenic spot coverage of toilets has increased from 15% to 93.5% (Fig 11), which greatly improves the quantity, quality and grade of tourist toilets, as well as the rationality and effectiveness of its spatial layout.

Tourism is an important driving force for the development of ethnic regions, but tourism activities themselves are full of mobility and uncertainty, especially for sensitive and complex ethnic areas. The single-threaded non-redundant system that single-sidedly emphasized economic efficiency in the past is obviously not suitable for this complex environment. We now need to have a certain degree of redundancy and space in this situation where efficiency may not be the highest, but it is sustainable. Equivalent to sustainable development, resilience theory focuses on the comprehensive development of economy, society and the environment. In fact, it is a theoretical model that emphasizes balance between different variables. This is consistent with the goal of paying more attention to fairness in addition to the optimization of facility layout. It helps researchers to have a more comprehensive understanding of the possible future prospects, and it is a bottom-up planning view from the perspective of tourists and residents, rather than simply representing the government and developers. From this perspective, in order to promote the sustainable development of tourism, this article aims to apply the concept of resilience in the field of tourism research, and provide a new perspective for the layout of sanitation facilities in tourism activities.

Resilience theory adopts a system view, solving the critical problem of how to make a system more capable of adapting to complex changes and responding to external shocks. The extension of the meaning of the tourist toilet means that it has already become a system, so this study focuses on improving the resilience of the tourist toilet system from the macro layout, which is a kind of resilience that concentrates more on materiality and structure. It should also be noted that tourist toilets represent a kind of living space, and it is necessary to enhance its social resilience. However, this article has insufficient consideration in this aspect. In the future, it should be improved from the perspective of relevant stakeholders to improve its social resilience. In addition, because the natural conditions of the scenic spots are different, for example, LBM scenic area is a virgin forest area, the topography and landforms have a greater impact on the layout of its tourist toilets, and the optimal plan provided by this paper is more ideal, which also leads regret. Furthermore, the micro-level resilient layout and design of tourist toilets, such as green roofs, rain barrels or reservoirs, are also directions that can be expanded in the future.

## 5. Conclusions

Achieving an optimal layout of tourist toilet is vitally important when it comes to meeting the public service needs of residents and tourists, enhancing the quality of tourism, maximizing local welfare as well as establishing a harmonious society. For those underdeveloped ethnic regions where tourism is a regional priority, the tourism environment is in urgent need of improvement.

Employing the term ‘tourist toilet’ in its broad sense, we analyzed the current situation of the layout of tourist toilets form the perspective of urban and scenic areas in Dunhua City. Our findings showed that tourist toilets in the urban area presents characteristics of quantity shortage, lower class, limited service scope, and extremely imbalanced distribution in new and old districts, while the distribution of tourist toilet resources in scenic areas is seriously polarized with large differences in density and quality. Besides, the layout ignored the external impact brought by changes in tourists.

In view of this, this study, for the first time, provides an empirical evidence for incorporating resilience theory into sanitation facility location decisions, approaching the gap of such research in the tourism field. We put forward reasonable locations and optimal layout schemes of tourist toilets in Dunhua by establishing AHP index, applying LA model and POI data crawling by Python crawler, as well as the statistics and remote sensing data etc. Correspondingly, the service scope of urban toilets has moderately overflowed into the relatively sparsely populated areas, while the resilience of toilets in the densely populated areas has been enhanced. The schemes also significantly improve the overall service capacity of toilets in scenic areas. Moreover, resilient toilets setting conforms to the seasonality and timeliness of local tourism activities. Thus, the system service could be as efficient as possible, improving the general quality of the toilet environment as a whole and achieving equitable access of toilets for both residents and tourists.

Under the macro background of China’s tourism toilet revolution, the optimal layout of tourist toilets with resilience is a promotion of the tourism development in underdeveloped ethnic regions, which can also drive their economic growth, ecology restoration, social progress and image establishment. This study also provides references for other countries, who are dedicated to promoting tourism sanitation and public health, and further achieving sustainable development.
